# The relationship between improved elderly nutritional risk index and short-term all-cause mortality in patients with urinary sepsis: a retrospective cohort study

**DOI:** 10.3389/fnut.2025.1700486

**Published:** 2026-01-12

**Authors:** Rui Liu, Wei Li, Xi Wei, Fa Sun, Tao Li

**Affiliations:** Department of Urology, Affiliated Hospital of Guizhou Medical University, Guiyang, China

**Keywords:** modified geriatric nutritional risk index, urosepsis, MIMIC-IV database, short-term mortality, risk prediction

## Abstract

**Background:**

The modified Geriatric Nutritional Risk Index (mGNRI) is a simple, objective tool for assessing malnutrition risk. Its potential utility in patients with urosepsis, however, remains insufficiently explored.

**Methods:**

We conducted a retrospective study of patients with sepsis secondary to urinary tract infections using data from the Medical Information Mart for Intensive Care (MIMIC-IV) database and a cohort from the Affiliated Hospital of Guizhou Medical University. The association between the mGNRI and short-term adverse outcomes was examined using restricted cubic spline (RCS) regression, multivariate Cox proportional hazards regression, Kaplan–Meier survival curves, and subgroup analyses. Furthermore, multivariate Cox regression was employed to evaluate the incremental predictive value of mGNRI when integrated with conventional critical illness scores.

**Results:**

This study included 1,875 patients with urosepsis. The 28-day ICU and in-hospital mortality rates were 15.5 and 14.3%, respectively. In fully adjusted models, both the continuous and categorical mGNRI were significantly associated with 28-day mortality. For each one-unit increase in the continuous mGNRI, the hazard ratios (HRs) for ICU and in-hospital mortality were 0.98 (95% CI: 0.97–0.99) and 0.98 (95% CI: 0.96–0.99), respectively. Similarly, for each one-standard deviation (SD) increase, the HRs were 0.83 (95% CI: 0.73–0.94) for ICU mortality and 0.81 (95% CI: 0.70–0.93) for in-hospital mortality. When using the no-risk group as a reference, the high-risk group exhibited significantly increased mortality, with HRs of 1.55 (95% CI: 1.07–2.24) for ICU death and 1.59 (95% CI: 1.08–2.32) for in-hospital death. RCS analysis revealed a negative linear relationship between the continuous mGNRI and mortality. Furthermore, subgroup and interaction analyses demonstrated that this association remained consistent across nearly all predefined subgroups. All findings were subsequently validated in an external, real-world cohort.

**Conclusion:**

Our findings indicate that the mGNRI serves as a significant inverse predictor of short-term mortality risk in patients with urosepsis. It demonstrates potential as a practical stratification tool to help clinicians identify high-risk patients early for targeted interventions.

## Background

Sepsis syndrome is a complex inflammatory response to infection, characterized by critical illness and high mortality, and has become a leading cause of death in non-cardiac intensive care units (ICUs) (1, 2). This syndrome can arise from infections at various sites, each with distinct pathophysiological features: pulmonary infections primarily involve alveolar-capillary barrier disruption and macrophage overactivation, often progressing to acute respiratory distress syndrome; abdominal infections are marked by bacterial translocation and mixed pathogen-associated molecular patterns, frequently leading to intra-abdominal hypertension; whereas urinary tract infections typically trigger systemic inflammation via Gram-negative bacterial endotoxins and early renal impairment (3–5). These source-specific pathological mechanisms—and their corresponding differences in treatment, highlight the importance of individualized, source-based precision medicine in sepsis management.

Genitourinary tract infections account for 9–31% of all sepsis cases, a condition specifically defined as urosepsis. As a severe complication of urinary tract infection (UTI), urosepsis poses a significant threat to individual health and imposes a substantial public health burden (6, 7). In recent years, the rising incidence of urinary tract diseases and related surgeries, coupled with increasing antimicrobial resistance, has further exacerbated the impact of urosepsis (3, 4). Therefore, investigating risk factors for UTI-related bloodstream infections and identifying biomarkers for early detection of high-risk populations are essential for strengthening preventive strategies and optimizing treatment. Previous studies have established a close relationship between patient nutritional status and the occurrence and prognosis of urosepsis (8, 9). Malnutrition, as indicated by specific nutritional metrics, is significantly associated with higher incidence and mortality risk in urosepsis, underscoring the importance of early nutritional assessment and intervention in clinical practice (8–11). However, existing nutritional screening tools are often difficult to implement widely due to their complexity and cumbersome criteria (12). There is an urgent need to develop a rapid, simple, and objective screening protocol to help healthcare providers promptly identify nutritional risks in ICU patients admitted with urosepsis.

The Geriatric Nutritional Risk Index (GNRI) is a simple and effective assessment tool that has been widely used for prognostic prediction in critically ill patients, such as those with severe pneumonia or cardiovascular events (13–17). It is calculated based on serum albumin and percentage of ideal body weight. However, when actual body weight exceeds the ideal value, the index sets the ideal weight percentage at 100%, limiting its accuracy in patients who may exhibit the “obesity paradox” (18). To address this limitation, a modified GNRI (mGNRI) was recently proposed, which adjusts the weighting of albumin to improve applicability and predictive performance in obese critically ill patients (18). Currently, the utility of mGNRI in prognostic assessment of urosepsis patients remains underexplored. This study aims to evaluate whether mGNRI can be used to predict clinical outcomes in this population.

## Methods and study population

### Data sources

This study was conducted using two retrospective cohorts. The internal training cohort was derived from the publicly available Critical Care Medicine Database, MIMIC-IV (19), maintained by the Massachusetts Institute of Technology. This database contains information from 196,527 adult patients admitted to the Beth Israel Deaconess Medical Center between 2008 and 2019. Access to the database was granted after approval; as the research involved only retrospective analysis of de-identified data, it was exempt from institutional review board (IRB) review. The external validation cohort comprised patients with urosepsis admitted to the Department of Urology and Critical Care Medicine at the Affiliated Hospital of Guizhou Medical University between January 2020 and January 2025. The construction of this cohort was implemented only after approval by the local hospital’s ethics committee.

### Study population

This study focused on patients admitted to the Intensive Care Unit (ICU) with sepsis secondary to a urinary tract infection (UTI). Patient identification followed established clinical criteria (20, 21). Sepsis was defined according to the Sepsis-3 consensus, requiring an increase of ≥2 points in the Sequential Organ Failure Assessment (SOFA) score from baseline. Patients with a primary admission diagnosis of UTI who met the Sepsis-3 criteria were enrolled in the UTI-sepsis cohort. The method for confirming UTI diagnosis differed between the two cohorts. For the internal cohort, identified from the MIMIC-IV database, diagnosis relied on ICD diagnostic codes. For the external cohort, UTI was confirmed within the first 24 h of admission based on typical clinical manifestations supported by laboratory evidence (pyuria and/or bacteriuria on urinalysis) and a positive urine culture. Importantly, a diagnosis of UTI was not contingent upon the administration of antibiotics. All enrolled patients met the following criteria: (1) age ≥18 years; (2) an ICU and total hospital stay exceeding 24 h; (3) availability of complete data for BMI and serum albumin levels; and (4) absence of known conditions that significantly affect serum albumin levels (e.g., malignancy), thereby excluding non-nutritional causes of hypoalbuminemia.

### Variable extraction

After obtaining the necessary permissions, data extraction was performed using PostgreSQL (version 13.7.2) and Navicat Premium (version 16.0) tools, in conjunction with Structured Query Language (SQL). The extracted variables were categorized into six groups: (1) demographic data; (2) comorbidities; (3) vital signs; (4) laboratory test indicators; (5) disease severity scores; and (6) administered treatments. Detailed descriptions of each variable are provided in Supplementary Tables S1, S2. For handling missing data, variables with missing values exceeding 30% were excluded from the analysis. For the remaining variables included in the analysis that still contained missing values, multiple imputation was performed using the “mice” package (v3.16.0) in R. The imputation model incorporated all variables used in the primary analysis: continuous variables requiring imputation, complete primary exposure variables (mGNRI and its components), the complete outcome variable (28-day mortality), and all other complete categorical and continuous covariates. This approach enhances the plausibility of the Missing at Random (MAR) assumption by leveraging all available information to predict missing values.

### Definition of nutritional status and endpoints

The mGNRI was derived from the original Geriatric Nutritional Risk Index (GNRI) as described by Hansen et al. (18). It was calculated using the following formula: mGNRI = [1.489 × ALB (g/L)] + [41.7 × (1–0.75 × |WLo - W| / WLo)], where: ALB is the serum albumin concentration in g/L. W is the patient’s actual body weight. WLo is the ideal body weight, calculated using the Lorentz formula, the Lorentz formula: For males: WLo (kg) = 50 + 0.91 × (height in cm - 152.4), For females: WLo (kg) = 45.5 + 0.91 × (height in cm - 152.4). To ensure biological plausibility, input data were cleaned prior to analysis. Body mass index (BMI) was constrained to a range of 10–100 kg/m^2^, and serum albumin was constrained to 1.0–6.0 g/dL to minimize the influence of extreme outliers on the stability of the results. For data visualization and comparative analysis, patients were categorized into four nutritional risk groups based on mGNRI quartiles: no risk (>84.15), low risk (77.25–84.15), moderate risk (70.22–77.25), and high risk (<70.22). This categorization was used for descriptive and exploratory purposes only and does not represent clinically validated risk thresholds. The primary outcome was 28-day all-cause mortality following ICU admission. The secondary outcome was in-hospital all-cause mortality during the same 28-day period.

### The correlation between mGNRI and short-term mortality in patients with urinary sepsis

To assess the association between the modified Geriatric Nutritional Risk Index (mGNRI) and the primary outcome, we constructed three sequentially adjusted Cox proportional hazards models: Model 1 was unadjusted; Model 2 was adjusted for demographics (age, sex, ethnicity, BMI); and Model 3 was further adjusted for clinically relevant covariates that showed significant differences between survivors and non-survivors, an approach consistent with prior studies (22–25). To control for multicollinearity, we calculated the Variance Inflation Factor (VIF) for all variables in Model 3 and excluded those with a VIF > 5. The final covariates retained in Model 3 included: Age, Gender, BMI, AKI, SOFA, APSIII, SAPSII, OASIS, CHARLSON, APACHEII, NBPS, RDW, RBC, WBC, AG, CO2, Fca, Lac, PH, Po2, INR, TB, UREA, VP, SA, GC, and CRRT. To examine the potential for a non-linear relationship, we used restricted cubic splines (RCS) with knots at the 5th, 35th, 65th, and 95th percentiles. The association between mGNRI levels and 28-day mortality was visualized using Kaplan–Meier survival curves. Finally, we conducted subgroup analyses and interaction tests to assess the potential influence of demographic characteristics and comorbidities on the primary association.

### Statistical analysis

Measurement data are described as mean ± standard deviation (SD). If normality and homogeneity of variance were satisfied, intergroup comparisons were made using Student’s t-test or analysis of variance (ANOVA). Count data are expressed as numbers (percentages), and intergroup comparisons were performed using Pearson’s chi-square test or Fisher’s exact test. All statistical analyses were performed using R software (version 4.5.1), and graphs were generated using the “ggplot2” package. A two-sided *p*-value < 0.05 was considered statistically significant.

## Results

### Baseline characteristics by nutritional status

This study ultimately included 1,875 patients with urosepsis who met the screening criteria. The mean age was 69.1 years, and 817 (43.6%) were male. Table 1 presents the baseline characteristics of the patients grouped by mGNRI. Analysis revealed that patients with lower mGNRI scores were older, had more comorbidities, and required more complex therapeutic interventions. The high-risk group showed more pronounced instability in vital signs such as heart rate, respiratory rate, and systolic blood pressure compared to the no-risk group. Significant differences were observed in numerous laboratory indicators between the groups: the high-risk group had higher levels of RDW, WBC, blood chloride, lactate, and blood urea nitrogen (BUN), but lower levels of HCT, hemoglobin, red blood cell count, total carbon dioxide combining power, serum ionized calcium, AST, and pH. Furthermore, clinical severity scores indicated a positive correlation between disease severity and mGNRI risk stratification. Mortality was significantly higher in the high-risk group compared to the no-risk group, specifically in terms of 28-day ICU mortality (22.8% vs. 10.4%, *p* < 0.001) and in-hospital mortality (21.7% vs. 9.36%, *p* < 0.001).

**Table 1:** Summary descriptive table by groups of mGNRI group.

Variable	ALL	No	Low	Moderate	High	*p* value
	*N* = 1875	*N* = 469	*N* = 469	*N* = 469	*N* = 469	
mGNRI	77.5 (9.93)	90.4 (5.30)	80.6 (1.93)	73.9 (2.04)	65.1 (4.07)	<0.001
Age	69.1 (15.1)	67.6 (15.7)	70.0 (15.1)	69.7 (14.4)	69.1 (14.9)	0.085
Gender	817 (43.6%)	196 (41.7%)	205 (43.9%)	209 (44.6%)	207 (44.1%)	0.817
Race	1,177 (62.8%)	281 (59.8%)	310 (66.4%)	300 (64.0%)	286 (61.0%)	0.149
BMI	29.9 (8.98)	30.9 (9.00)	30.1 (8.55)	29.7 (8.96)	29.1 (9.34)	0.020
Hyp	707 (37.7%)	195 (41.5%)	173 (37.0%)	176 (37.5%)	163 (34.8%)	0.195
AKI	1,173 (62.6%)	266 (56.6%)	276 (59.1%)	293 (62.5%)	338 (72.1%)	<0.001
CKD	510 (27.2%)	122 (26.0%)	150 (32.1%)	116 (24.7%)	122 (26.0%)	0.049
DM	681 (36.3%)	162 (34.5%)	191 (40.9%)	162 (34.5%)	166 (35.4%)	0.125
HF	723 (38.6%)	192 (40.9%)	190 (40.7%)	173 (36.9%)	168 (35.8%)	0.265
MI	200 (10.7%)	56 (11.9%)	47 (10.1%)	49 (10.4%)	48 (10.2%)	0.786
IHD	709 (37.8%)	175 (37.2%)	169 (36.2%)	190 (40.5%)	175 (37.3%)	0.553
COPD	332 (17.7%)	81 (17.2%)	93 (19.9%)	82 (17.5%)	76 (16.2%)	0.498
SOFA	7.05 (3.76)	6.35 (3.69)	6.72 (3.59)	7.30 (3.76)	7.84 (3.82)	<0.001
APSIII	58.7 (22.3)	52.4 (21.2)	56.0 (20.2)	60.2 (21.5)	66.0 (23.7)	<0.001
SIRS	2.83 (0.91)	2.66 (0.93)	2.80 (0.92)	2.90 (0.88)	2.96 (0.88)	<0.001
SAPSII	45.0 (14.0)	41.6 (13.4)	44.0 (13.7)	46.1 (13.5)	48.4 (14.6)	<0.001
OASIS	36.7 (8.43)	34.6 (8.25)	36.8 (8.74)	37.7 (7.79)	38.0 (8.52)	<0.001
CHARLSON	6.06 (2.86)	5.85 (2.79)	6.23 (2.92)	5.99 (2.90)	6.19 (2.82)	0.135
APACHEII	21.8 (7.34)	20.5 (7.33)	21.2 (7.12)	22.2 (7.12)	23.5 (7.46)	<0.001
HR	91.9 (21.8)	88.4 (20.8)	90.9 (21.8)	92.0 (20.6)	96.4 (23.1)	<0.001
NBPS	121 (26.2)	125 (26.6)	121 (25.8)	120 (25.0)	117 (26.8)	<0.001
NBPD	68.5 (20.3)	69.7 (19.9)	69.0 (19.2)	67.8 (19.8)	67.3 (22.0)	0.244
RR	20.1 (6.54)	19.6 (6.20)	19.7 (6.53)	20.0 (5.99)	21.3 (7.25)	0.001
Spo2	96.5 (4.76)	96.3 (4.74)	96.8 (4.11)	96.5 (5.25)	96.5 (4.84)	0.297
HCT	31.8 (6.70)	33.3 (6.88)	32.2 (6.64)	31.4 (6.30)	30.1 (6.56)	<0.001
Hb	10.3 (2.24)	10.8 (2.32)	10.5 (2.22)	10.2 (2.05)	9.74 (2.22)	<0.001
PLT	204 (116)	200 (99.3)	204 (110)	205 (128)	207 (126)	0.724
RDW	15.9 (2.65)	15.7 (2.76)	15.7 (2.50)	16.0 (2.59)	16.4 (2.69)	<0.001
RBC	3.46 (0.79)	3.65 (0.85)	3.52 (0.77)	3.41 (0.73)	3.27 (0.76)	<0.001
WBC	13.9 (11.7)	12.7 (10.6)	13.9 (13.1)	13.5 (7.96)	15.3 (14.0)	0.012
ALB	2.92 (0.60)	3.60 (0.40)	3.06 (0.29)	2.71 (0.33)	2.29 (0.39)	<0.001
AG	15.6 (4.84)	15.9 (4.92)	15.5 (4.68)	15.6 (4.82)	15.6 (4.95)	0.664
Cl	104 (7.82)	102 (6.96)	104 (7.85)	105 (7.78)	105 (8.46)	<0.001
Glu	157 (84.9)	155 (77.6)	157 (76.2)	158 (88.9)	159 (95.4)	0.930
K	4.24 (0.82)	4.23 (0.85)	4.22 (0.76)	4.27 (0.79)	4.23 (0.86)	0.748
TCO2	23.9 (6.39)	24.7 (6.79)	24.6 (6.19)	23.2 (5.99)	23.0 (6.42)	<0.001
FCa	1.11 (0.13)	1.12 (0.12)	1.11 (0.13)	1.10 (0.13)	1.09 (0.13)	<0.001
Lac	2.43 (2.05)	2.28 (1.91)	2.27 (1.93)	2.45 (2.00)	2.72 (2.31)	0.004
PCo2	42.0 (12.7)	42.4 (13.8)	42.9 (12.6)	41.3 (11.4)	41.4 (12.7)	0.154
PH	7.35 (0.11)	7.36 (0.10)	7.35 (0.11)	7.34 (0.11)	7.34 (0.12)	0.001
Po2	128 (103)	134 (108)	130 (107)	131 (101)	118 (96.2)	0.061
INR	1.66 (1.03)	1.63 (0.87)	1.64 (1.01)	1.69 (1.19)	1.69 (1.04)	0.648
PT	18.0 (10.5)	17.6 (8.98)	17.8 (10.4)	18.3 (11.8)	18.3 (10.6)	0.672
APTT	40.4 (25.3)	40.1 (25.7)	39.6 (24.3)	41.7 (27.8)	40.4 (23.3)	0.659
ALT	153 (687)	228 (1075)	108 (327)	150 (647)	124 (444)	0.102
AST	269 (1229)	370 (1689)	166 (550)	291 (1371)	250 (994)	0.022
TB	2.10 (4.86)	2.36 (5.81)	1.92 (4.75)	2.16 (4.87)	1.95 (3.81)	0.532
CRE	1.81 (1.73)	1.70 (1.65)	1.81 (1.78)	1.88 (1.72)	1.85 (1.77)	0.380
UREA	36.1 (28.9)	31.9 (27.0)	35.5 (29.7)	36.5 (25.8)	40.4 (32.0)	<0.001
LDH	598 (1389)	623 (1549)	544 (1252)	617 (1347)	606 (1392)	0.781
SA	1,529 (81.5%)	375 (79.8%)	382 (81.8%)	386 (82.3%)	386 (82.3%)	0.719
VP	1,336 (71.3%)	293 (62.3%)	333 (71.3%)	331 (70.6%)	379 (80.8%)	<0.001
GC	654 (34.9%)	162 (34.5%)	153 (32.8%)	163 (34.8%)	176 (37.5%)	0.492
Ventilation	1742 (92.9%)	446 (94.9%)	444 (95.1%)	431 (91.9%)	421 (89.8%)	0.003
CRRT	270 (14.4%)	60 (12.8%)	54 (11.6%)	76 (16.2%)	80 (17.1%)	0.046
Hosp day	21.4 (21.1)	20.4 (18.6)	21.0 (22.6)	21.0 (19.4)	23.3 (23.3)	0.197
ICU day	9.17 (9.94)	9.40 (9.80)	8.81 (9.81)	9.19 (9.72)	9.29 (10.4)	0.812
Hosp dead	269 (14.3%)	44 (9.36%)	57 (12.2%)	66 (14.1%)	102 (21.7%)	<0.001
ICU dead	290 (15.5%)	49 (10.4%)	62 (13.3%)	72 (15.4%)	107 (22.8%)	<0.001

### Association between mGNRI and short-term mortality in Urosepsis patients

As shown in Table 2, after adjusting for all potential confounders, each 1-unit increase in the mGNRI score was associated with a 2% reduction in 28-day ICU mortality (HR = 0.98, 95% CI: 0.97–0.99, *p* = 0.005). Each 1-standard deviation (SD) increase was associated with a more substantial 17% reduction (HR = 0.83, 95% CI: 0.73–0.94, p = 0.005). Furthermore, the high-risk group had a 55% higher mortality risk compared to the no-risk group (HR = 1.55, 95% CI: 1.07–2.24, *p* = 0.022). These associations were consistent for in-hospital mortality (Table 3). Each 1-unit increase in the mGNRI corresponded to a 2% risk reduction (HR = 0.98, 95% CI: 0.96–0.99, *p* = 0.002), and each 1-SD increase was associated with a 19% reduction (HR = 0.81, 95% CI: 0.70–0.93, *p* = 0.005). The high-risk group demonstrated a 59% increased risk of in-hospital mortality compared to the no-risk group (HR = 1.59, 95% CI: 1.08–2.32, *p* = 0.017).

**Table 2:** The relationship between mGNRI and short-term mortality rate in ICU (internal discovery queue).

Characteristic	Model 1			Model 2			Model 3		
	HR	95% CI	*p*-value	HR	95% CI	*p*-value	HR	95% CI	*p*-value
mGNRI	0.97	0.96, 0.98	<0.001	0.97	0.96, 0.98	<0.001	0.98	0.97, 0.99	0.005
mGNRI_std	0.74	0.66, 0.83	<0.001	0.76	0.67, 0.85	<0.001	0.83	0.73, 0.94	0.005
mGNRI group									
No	Ref	Ref		Ref	Ref		Ref	Ref	
Low	1.37	0.95, 2.00	0.10	1.32	0.91, 1.93	0.14	1.23	0.84, 1.81	0.3
Moderate	1.51	1.05, 2.16	0.026	1.38	0.96, 1.98	0.082	1.28	0.88, 1.85	0.2
High	2.21	1.58, 3.11	<0.001	2.03	1.44, 2.86	<0.001	1.55	1.07, 2.24	0.022

CI, Confidence Interval; HR, Hazard Ratio.

**Table 3:** The relationship between mGNRI and short-term mortality rate in hospital (internal discovery queue).

Characteristic	Model 1			Model 2			Model 3		
	HR	95% CI	*p*-value	HR	95% CI	*p*-value	HR	95% CI	*p*-value
mGNRI	0.97	0.96, 0.98	<0.001	0.97	0.96, 0.98	<0.001	0.98	0.97, 0.99	0.002
mGNRI_std	0.72	0.64, 0.81	<0.001	0.75	0.66, 0.85	<0.001	0.81	0.70, 0.93	0.002
mGNRI group									
No	Ref	Ref		Ref	Ref		Ref	Ref	
Low	1.32	0.89, 1.96	0.2	1.25	0.84, 1.86	0.3	1.11	0.74, 1.66	0.6
Moderate	1.52	1.04, 2.22	0.032	1.38	0.94, 2.01	0.10	1.22	0.82, 1.80	0.3
High	2.22	1.56, 3.16	<0.001	1.99	1.39, 2.85	<0.001	1.59	1.08, 2.32	0.017

CI, Confidence Interval; HR, Hazard Ratio.

**Figure 1:**
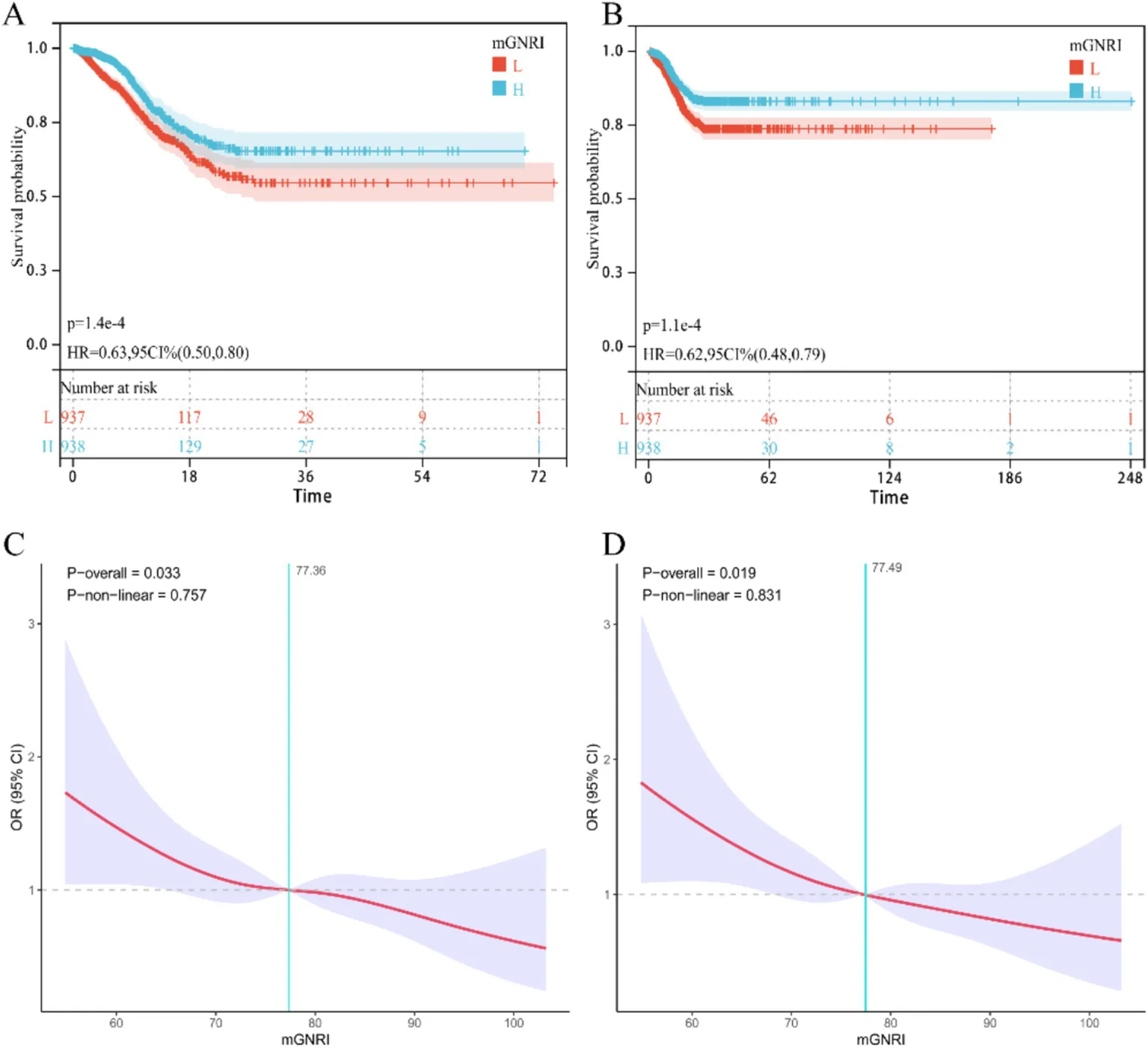
The relationship between mGNRI and short-term mortality rates in ICU and Hosp. **(A,B)** KM survival curve. **(A)** mGNRI and ICU short-term mortality rate, **(B)** mGNRI and Hosp short-term mortality rate; **(C,D)** RCS curve. **(C)** mGNRI and ICU short-term mortality rate, **(D)** mGNRI and Hosp short-term mortality rate. L, low; H, high.

**Figure 2:**
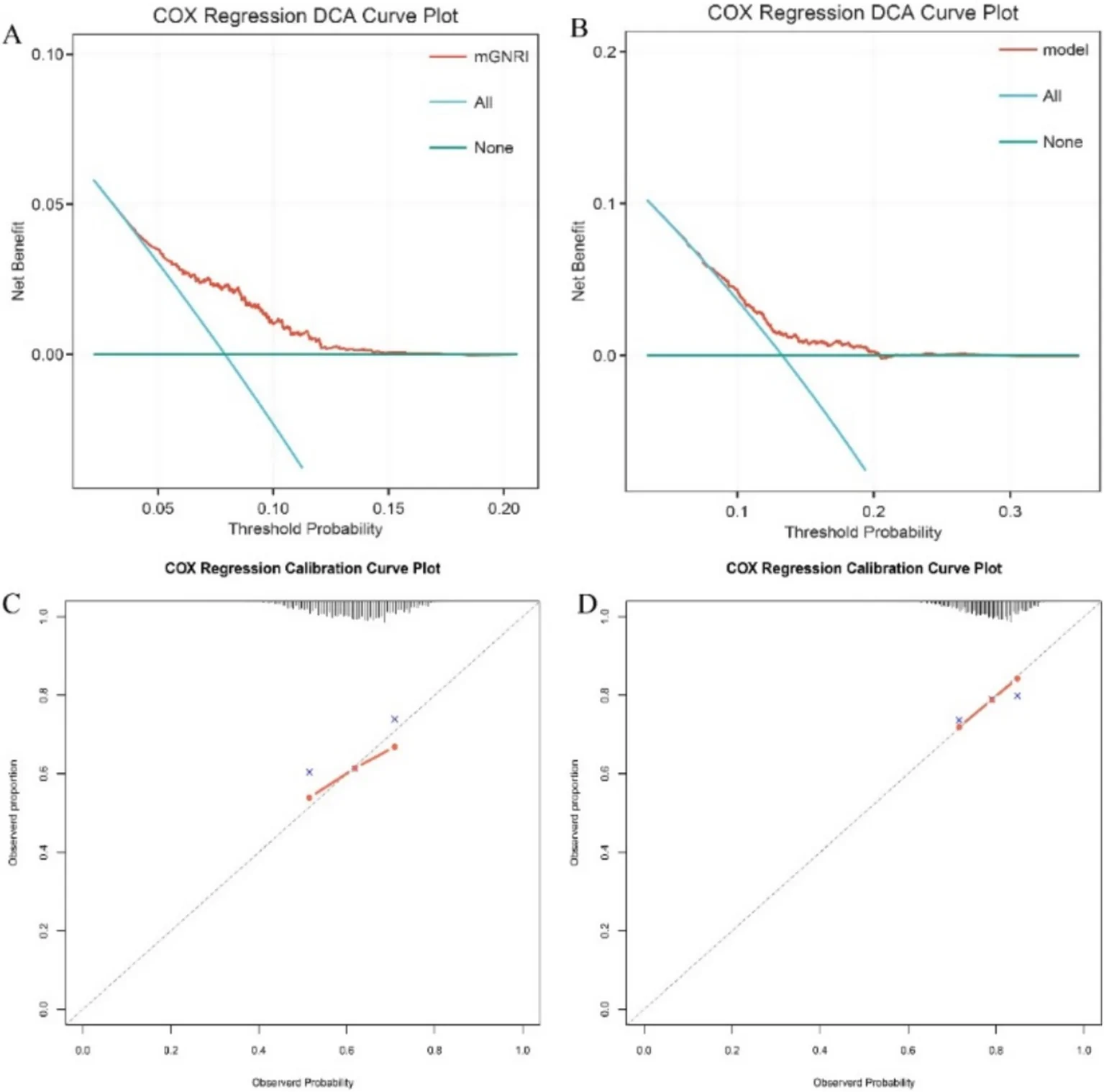
The results of decision curve and correction curve. **(A,B)** Decision curve; **(A)** mGNRI and ICU short-term mortality rate; **(B)** mGNRI and Hosp short-term mortality rate; **(C,D)** correction curve; **(C)** mGNRI and ICU short-term mortality rate; **(D)** mGNRI and Hosp short-term mortality rate.

RCS analysis demonstrated a significant inverse linear association between mGNRI and short-term mortality (*p* for overall association <0.05; *p* for non-linearity >0.05) (Figures 1A,B). Kaplan–Meier curves further confirmed that groups with higher mGNRI scores had significantly better survival outcomes (Figures 1C,D). Decision Curve Analysis (DCA) indicated that the model provided a superior net clinical benefit across a wide range of decision thresholds when compared to the strategies of intervening on all or no patients (Figures 2A,B). Furthermore, calibration curves confirmed that the mortality risk predicted by mGNRI was accurate and reliable, particularly for high-risk populations (Figures 2C,D).

### Incremental effect of mGNRI

**Figure 3:**
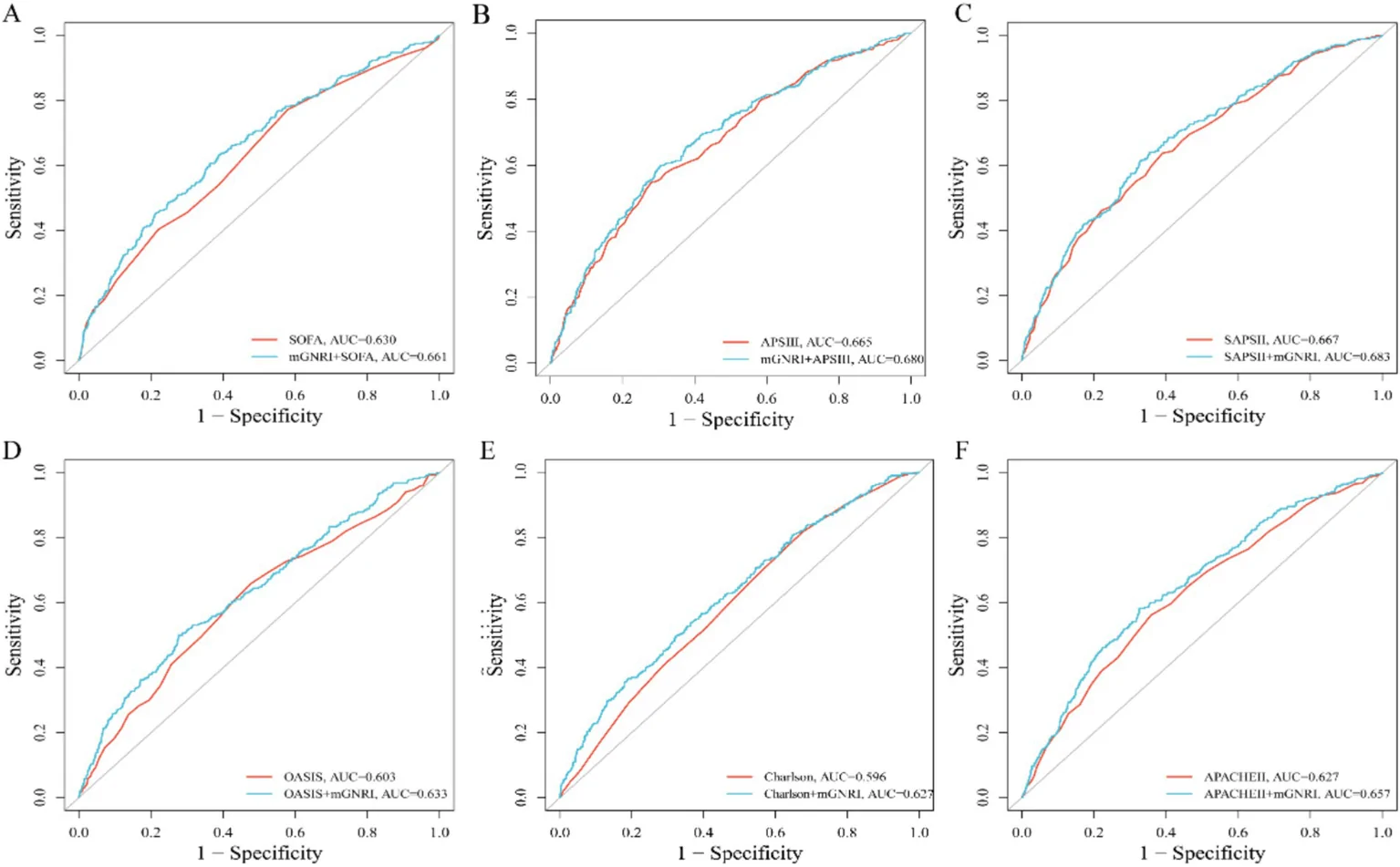
The incremental effect of mGNRI on the predictive performance of traditional severity scores. **(A–F)** ROC curve: **(A)** The incremental effect of mGNRI on SOFA; **(B)** the incremental effect of mGNRI on APSIII; **(C)** the incremental effect of mGNRI on SAPSII; **(D)** the incremental effect of mGNRI on OASIS; **(E)** the incremental effect of mGNRI on Charison; **(F)** the incremental effect of mGNRI on APACHEII.

To evaluate the additive value of the mGNRI, we assessed its combination with six established severity scores (SOFA, APS III, SAPS II, OASIS, CHARLSON, APACHE II) for predicting 28-day ICU mortality. The inclusion of the mGNRI consistently improved the predictive accuracy of all models (Figures 3A–F). DeLong tests confirmed that these improvements were statistically significant for each score (e.g., SOFA: AUC 0.630 to 0.691, *p* < 0.001; APS III: 0.665 to 0.680, *p* = 0.047). These results confirm that the incremental prognostic value provided by the mGNRI is robust and not due to chance.

### Subgroup and interaction analysis

To investigate whether demographic characteristics and comorbidities influence the association between mGNRI and 28-day ICU/in-hospital mortality, subgroup analyses and interaction tests were conducted. The fully adjusted models revealed some variation in the association between mGNRI and short-term mortality among urosepsis patients across different populations: the association remained consistent and significant in elderly, non-White, and female patients, but did not reach statistical significance in younger, White, and male patients. Moreover, mGNRI still showed a significant association with mortality risk in patients with hypertension, chronic kidney disease, diabetes, or ischemic heart disease. However, interaction tests did not reveal significant interaction effects between mGNRI and any of the aforementioned subgroup variables (all *p*-values > 0.05).

### Sensitivity analysis

Recognizing that albumin infusion and CRRT can significantly influence serum albumin levels, we performed a sensitivity analysis to test the robustness of our primary findings. We excluded patients who received albumin (*n* = 21) or CRRT (*n* = 17) within the first 24 h of ICU admission from the main cohort (*n* = 1875), creating a more homogeneous analysis cohort (*n* = 1837). Subsequent multivariate COX regression in this subgroup confirmed the protective association of mGNRI. For each 1-standard deviation (SD) increase in mGNRI, the adjusted Hazard Ratio (HR) was 0.83 (95% CI: 0.72–0.94, *p* = 0.005) for 28-day ICU mortality and 0.81 (95% CI: 0.70–0.92, *p* = 0.002) for 28-day in-hospital mortality. The direction and magnitude of this protective association were highly consistent with the results from our primary analysis. This consistency strengthens the conclusion that our main finding is robust and not substantially confounded by these specific, early interventions.

### Preliminary validation in real-world queues

**Figure 4:**
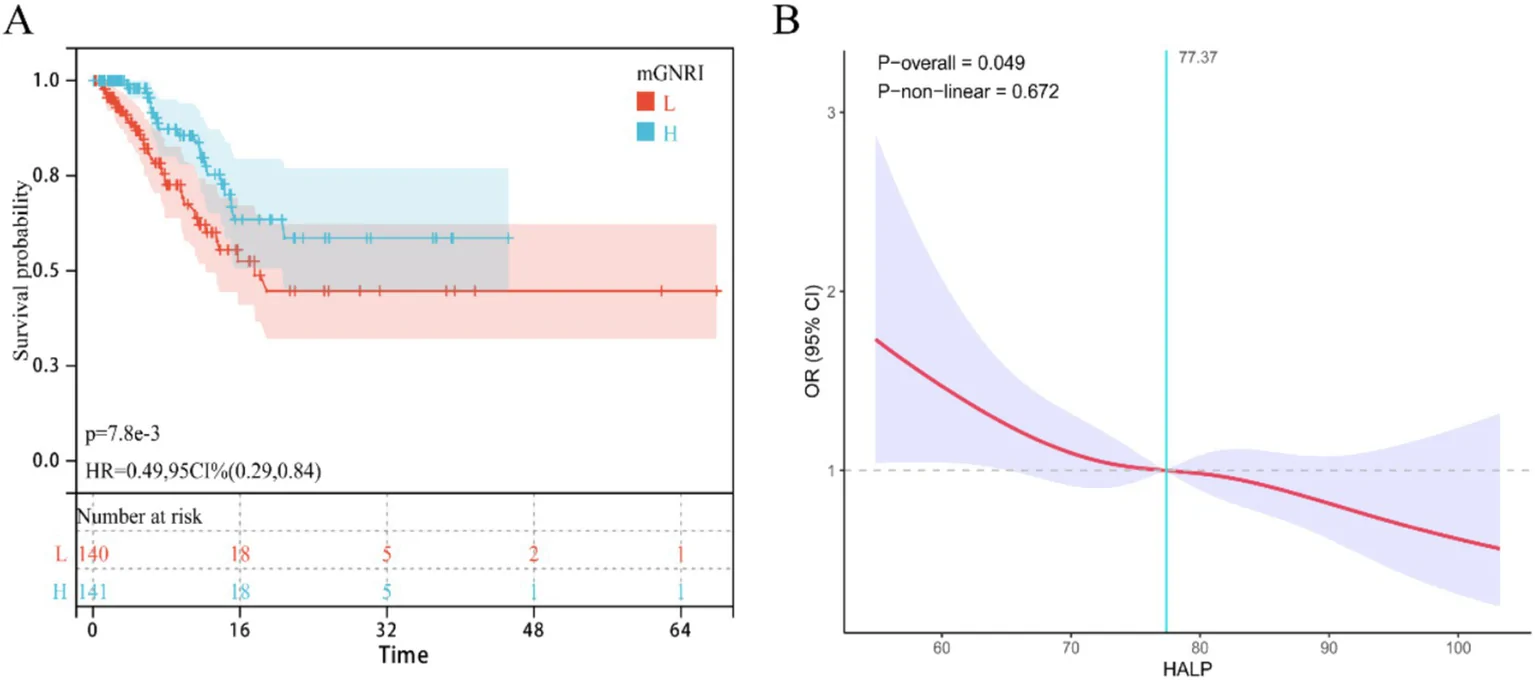
The relationship between mGNRI and short-term mortality rates in ICU (external verification queue). **(A)** KM survival curve of mGNRI and short-term ICU mortality; **(B)** RCS curves of mGNRI and short-term ICU mortality.

The external validation cohort included 281 patients with urosepsis (28-day mortality = 21.0%). In the Cox regression model adjusted for all potential covariates, a higher mGNRI score was significantly associated with a reduced risk of 28-day mortality (HR = 0.97; 95% CI: 0.94–1.00, *p* = 0.036). Subsequently, Kaplan–Meier analysis indicated that patients with mGNRI scores above the cohort median had a significantly lower mortality risk (log-rank *p* = 0.008; HR = 0.49, 95% CI: 0.29–0.84; Figure 4A). Furthermore, the RCS curve confirmed a significant dose–response relationship between mGNRI and mortality (*p* = 0.048; Figure 4B).

## Discussion

Urosepsis, a critical complication of urinary tract infection, has shown a consistent upward trend in incidence in recent years. This increase is closely linked to the rising number of urinary system diseases and surgical procedures, as well as increasing microbial antibiotic resistance, posing significant challenges to both individual patients and public health systems (1–4). Previous research has emphasized the importance of finding reliable and easily implementable biomarkers to improve the prognosis of these patients (1–7, 26–28). This study is the first to reveal a significant association between mGNRI and adverse outcomes in patients with urosepsis. The results indicate that a lower mGNRI is an independent risk factor for all-cause mortality both within the ICU and during hospitalization at 28 days. We further identified a linear, inverse dose–response relationship between mGNRI and early mortality in this population. Notably, mortality was significantly higher in the high-risk nutritional group compared to the no-risk group after stratification. Finally, subgroup and interaction analyses suggested that this association remained consistent across various demographic characteristics and comorbid conditions, indicating robust findings.

The GNRI is a simple and effective nutritional assessment tool widely used for screening malnutrition risk and predicting clinical prognosis in elderly patients (13). Numerous studies have affirmed its predictive value in ICU settings, where low GNRI scores are strongly associated with mortality and poor outcomes in various critical conditions, including sepsis, trauma, and acute kidney injury (14–17). However, the traditional GNRI has a theoretical flaw: when a patient’s actual weight exceeds their ideal weight, the weight ratio is set to an upper limit of 100%, preventing effective identification of individuals exhibiting the “obesity paradox” (concurrent overweight and malnutrition) (18). To address this issue, researchers recently proposed a modified version, the mGNRI. This adjusted index optimizes the weighting of albumin, allowing for a more reasonable reflection of the nutritional status of obese patients and demonstrating better predictive performance in critically ill populations (18). Consistent with advancements in the field, our study also confirms a significant association between mGNRI and adverse prognosis in patients with urosepsis.

Malnutrition, a state where energy intake fails to meet the body’s physiological needs, is closely related to the occurrence and progression of various severe diseases, including sepsis (29–31). The underlying mechanisms of this association are multifactorial, with the inflammatory response considered a core intermediary link (32, 33). Extensive experimental research indicates that an excessively activated immune-inflammatory response, followed by a compensatory anti-inflammatory response and immunosuppression, collectively form the central pathological process of sepsis development and progression 1 (34–36). A clear bidirectional interaction exists between malnutrition and inflammation: on one hand, hypoalbuminemia, a marker of malnutrition, is associated with a systemic hyperinflammatory state (36, 37); on the other hand, persistent inflammation can suppress albumin synthesis, further exacerbating malnutrition and creating a vicious cycle (38, 39). Furthermore, malnutrition can trigger processes such as oxidative stress, lipid peroxidation, immune dysregulation, and programmed cell death (40, 41). These mechanisms collectively exacerbate tissue and organ damage in sepsis (42).

Beyond the mechanisms shared with general sepsis, malnutrition may contribute to the onset and progression of urosepsis through several distinct pathways. Firstly, malnutrition can directly compromise the local defensive barrier of the urinary tract. The urothelium is coated by a protective layer of glycoproteins, the synthesis of which depends on adequate nutrition. Under malnutrition, a deficiency in essential nutrients can lead to the thinning or impairment of this layer, facilitating increased bacterial adhesion to the urothelial cells (43, 44). Secondly, previous research indicates that deficiencies in specific nutrients can enhance the virulence of uropathogenic *Escherichia coli*, thereby aggravating the infection (45). Thirdly, the normal urinary pH is a key factor influencing bacterial invasiveness. Malnutrition is often associated with metabolic disturbances that can lead to abnormal urinary acidity, potentially altering the environment in a way that favors bacterial colonization (46, 47). Finally, malnourished patients frequently have a higher burden of urinary calculi (stones). These stones can act as a reservoir for pathogens and cause urinary tract obstruction, which is a well-established primary risk factor for the development and exacerbation of urosepsis (5, 48).

The results of the subgroup analysis indicated that the strength of the association between mGNRI and short-term mortality in urosepsis patients varied depending on demographic characteristics and comorbidities. One possible explanation is that the mGNRI was originally designed to assess nutritional risk in the elderly; hence, it might have greater discriminative power in older populations or those with chronic wasting diseases. Although the interaction analysis did not reveal significant interaction effects between demographic variables and mGNRI, we speculate that the limited sample size within the current subgroups might have reduced statistical power. Therefore, larger-scale prospective cohort studies are needed in the future to further validate these associations and their potential influencing factors.

Despite confirming the independent prognostic value of the mGNRI for short-term outcomes in patients with urosepsis, this study has several limitations. First, its retrospective design precludes the establishment of causality between the mGNRI and mortality. Second, the nutritional assessment was based solely on data from the first 24 h of admission. This single-timepoint measurement cannot capture dynamic nutritional status throughout the ICU stay or fully represent a patient’s long-term nutritional history, particularly during recovery. Therefore, future research should employ Group-Based Trajectory Modeling to investigate the association between the dynamic trajectories of mGNRI and short-term mortality in patients with urosepsis (49). Third, our study is limited by the absence of certain key details in the database, such as specific urological procedures, causative bacterial pathogens, and information on mixed infections during hospitalization. These unmeasured variables represent potential confounders that could influence the reliability of our conclusions. Fourth, to maintain baseline comparability, we excluded patients who experienced acute events (e.g., major hemorrhage or cardiac arrest) or had missing key variables within a dual 24-h threshold. While this approach is supported by prior literature (50–53), it may introduce immortal time bias by systematically excluding the most critically ill patients, potentially leading to an underestimation of true mortality and a distortion of predictor-outcome associations. Fifth, the generalizability of our findings is limited by the single-center design and modest sample size. Furthermore, potential temporal shifts in clinical care underscore the need for larger, multi-center, prospective studies to validate the prognostic utility and clinical applicability of the mGNRI in this patient population. Finally, although the covariates for our primary model were selected based on established literature, this strategy is susceptible to bias. Future confirmatory studies should pre-specify covariates *a priori*, guided by robust clinical knowledge and causal diagrams (DAGs).

## Conclusion

The findings of this study indicate that the mGNRI serves as a significant inverse predictor of short-term mortality risk in patients with urosepsis. It demonstrates potential as a practical stratification tool to help clinicians identify high-risk patients early for targeted interventions.

## Data Availability

The original contributions presented in the study are included in the article/Supplementary material, further inquiries can be directed to the corresponding author/s.
